# Surgical repair of rectovaginal fistula by combined transanal and transvaginal endoscopy: a case report

**DOI:** 10.3389/fgstr.2024.1364379

**Published:** 2024-05-08

**Authors:** Tian He, Wen Zhang, Nian-fen Mao, Xuan Bai, Lin Zhao, Ke-lin Yue, Guo-qing Yang, Chun-mei Rao, Jing Wang, Ping Wan, Qiang Guo, Zan Zuo

**Affiliations:** ^1^ Clinical Center of Digestive Endoscopy of Yunnan Province, Department of Gastroenterology, The First People’s Hospital of Yunnan Province, Kunming, Yunnan, China; ^2^ Medical College, Kunming University of Science and Technology, Kunming, Yunnan, China; ^3^ Internal Medicine, Yunnan University of Traditional Chinese Medicine, Kunming, Yunnan, China

**Keywords:** rectovaginal fistula (RVF), transanal endoscopic surgery (TES), transvaginal endoscopic surgery, case report, new technology

## Abstract

The common causes of rectovaginal fistula include obstetric trauma, local infection, and rectal surgery, while rectovaginal fistula following hemorrhoid surgery is extremely rare. Rectovaginal fistulae (RVF) rarely heal without intervention. Surgical treatment is usually performed, but the optimal surgical method remains controversial. The patient was a 37-year-old woman who was transferred to our hospital due to an unsuccessful repair of a rectovaginal fistula after hemorrhoid surgery in a local hospital. The next day after admission, she had prophylactic ileostomy, fecal diversion and combined treatment with cephalosporin antibiotic to create a clean postoperative area. However, there was still fecal outflow from the vagina, with no significant reduction in excretion compared to previous surgery. Digestive endoscopy confirmed a failure of the repair for rectovaginal fistula. Therefore, preventive ileostomy was continued to reduce the accumulation of bacteria in the fistula and control the inflammation. After 8 weeks, the endoscopic fistula inflammation disappeared, and the condition of endoscopic surgery was considered to be mature. Subsequently, a new surgical method combining transanal endoscopy and transvaginal endoscopy was performed. After 12 weeks of surgery, a follow-up endoscopic examination showed that the fistula had been repaired and healed. During the 1-year follow-up, no serious complication was encountered, no recurrence was found, and the repair effect was satisfactory. In conclusion, a new technique combining transanal endoscopy and transvaginal endoscopy can effectively be used for the surgical repair of rectovaginal fistula after a hemorrhoid operation.

## 1 Clinical background

Clinically, rectovaginal fistulas are relatively rare. The common causes are obstetric trauma, local infection, and rectal surgery. Rectovaginal fistula is a pathological channel composed of epithelial tissue between the anterior wall of the rectum and the posterior wall of the vagina, which is reported to account for approximately 5% of all anorectal fistulas (1). Because of the particularity of the local anatomy, the complex etiology, and the differences between individual patients, treatment remains extremely challenging. In addition, the formation of scars and the sclerosis of surrounding tissues after the procedure for prolapse and hemorrhoids (PPH) combined with injection sclerotherapy increase the difficulty in repairing the rectovaginal fistula. Therefore, for this patient with rectovaginal fistula after hemorrhoid surgery, transanal endoscopy and vaginal endoscopy were performed for treatment, followed by the creation of the mucosal pinhole under a digestive endoscope, the electrocauterization of the mucosal tissue in the fistula, and stoma clamping with nylon ring-assisted through-the-scope-clip (TTSC) “purse suture’” clipping (2). This new method achieved a good therapeutic effect and is reported in the following paragraphs.

## 2 Case presentation

The patient was a 37-year-old woman who was hospitalized in a local hospital due to hemorrhoids and underwent PPH combined with injection sclerotherapy. On the fourth day after operation, rectovaginal fistula was found due to vaginal discharge, fecal discharge, and bloody purulent secretions. Thus, the patient underwent transvaginal fistula resection and layered suture repair, and was treated with levofloxacin for anti-infection in that hospital. Three days after operation, there were still bloody purulent secretions and fecal discharge in the vagina; thus, the patient was transferred to our First People’s Hospital of Yunnan Province.

The laboratory examination showed that the white blood cell level was 12 × 10^9^/L, the neutrophil level was 9.6 × 10^9^/L, the high-sensitivity C-reactive protein level was 126 mg/L, and the procalcitonin level was 1.26 ng/mL, indicating that there was constant infection and inflammation. Then, CT evaluation of the rectum showed a linear dense shadow, with no obvious clear fistula. Considering that there was a reduction of vaginal discharge of the patient, and the recovery time was short postoperatively, in addition to the imageological and laboratory examinations, the doctors decided to perform prophylactic ileostomy, fecal diversion and combined treatment with cephalosporin antibiotics on the patient. However, after the 7-day treatment, even though no purulent excretion was observed, there was still fecal outflow from the vagina, with no significant change in the amount of discharge by dynamic monitoring. Digestive endoscopy revealed the failure of the surgical repair as mucosal inflammation was found at both the stoma and the margin of the rectovaginal fistula, while the mucosa in the fistula cavity had been repaired and healed to form a channel. Considering the postoperative short period and inflammation at the edge of the fistula, the timing of this hospitalization was not suitable for endoscopic intervention. Preventive ileostomy and fecal diversion treatment were continued for 8 weeks (4 weeks as required), then the inflammation at the fistula stoma disappeared under endoscopic reexamination, indicating a fair-enough surgical condition; thus, the surgical repair of the rectovaginal fistula by combined transanal and transvaginal endoscopy was carried out. Written informed consent was obtained from the patient for the publication of this case report. The case report was reviewed and approved by the Institutional Review Board of the First People’s Hospital of Yunnan Province (Approval No. KHLL2023-KY119).

## 3 New technique for treatment

Rectovaginal fistulae are relatively uncommon. Surgical approaches for repairing these fistulae include the transvaginal, transrectal, transperineal, or transabdominal (cesarean or laparoscopic) routes, depending on the complexity of the fistula and the presence of associated sphincter injury. Following the PPH for rectal mucosa combined with the sclerotherapy by injection of sclerosing agents, the sclerotic tissue and fibrotic scar repair increased the difficulty in RVF treatment. This study describes a new method for repairing rectovaginal fistulae via dual ano-vaginal access with gastrointestinal endoscopic argon plasma coagulation (APC) combined with clip closure.

**Figure 1:**
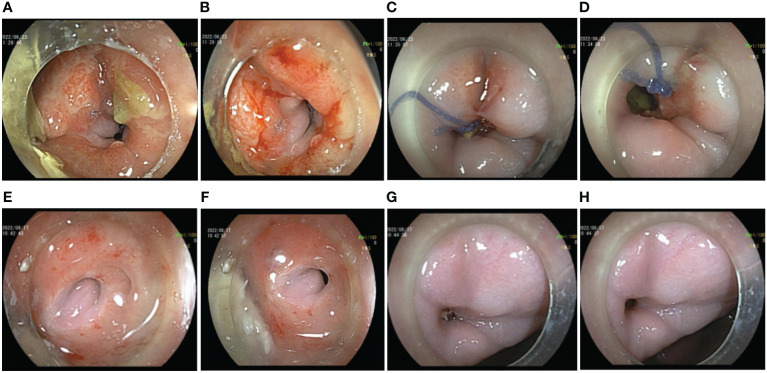
Preoperative auxiliary examinations of the patient. **(A, B)** Endoscopic manifestations of rectal fistula on the first examination. **(C, D)** Endoscopic manifestations of vaginal fistula on the first examination. **(E, F)** Endoscopic manifestations of rectal fistula 1 month after ileostomy. **(G, H)** Endoscopic manifestations of vaginal fistula 1 month after ileostomy.

With comprehensive evaluation, the admitted patient was treated with prophylactic ileostomy and defecation diversion combined with cephalosporin antibiotics. After 7 days, there was still fecal outflow from the vagina. The colon endoscopy showed a fistula with a transverse diameter of approximately 10 mm in the anterior wall of the rectum approximately 5 cm from the anus, with significant inflammation, mucosal congestion, and edema (**Figures 1A, B**). The vaginal endoscopy showed a fistula in the posterior wall of the vagina, which was connected with the rectal fistula, and the suture was visualized (**Figures 1C, D**). A sample was collected from the edge of the fistula for histopathological examination, showing no sign of malignancy. Because only 11 days have passed since the vaginal fistula resection and layered suture repair treatment of the patient, the recovery time of the tissue was short, and the edge of the fistula was inflammatory, it was not the right time for endoscopic intervention; thus, the preventive ileostomy and defecation diversion treatment were continued. Eight weeks later, a reexamination of digestive endoscopy revealed that the mucosal inflammation at the margin of fistula was significantly relieved, and the fistula mucosa showed white scar-like changes, even though the existence of the rectovaginal fistula was still visible (**Figures 1E–H**). Therefore, endoscopic rectovaginal fistula repair was carried out.

**Figure 2:**
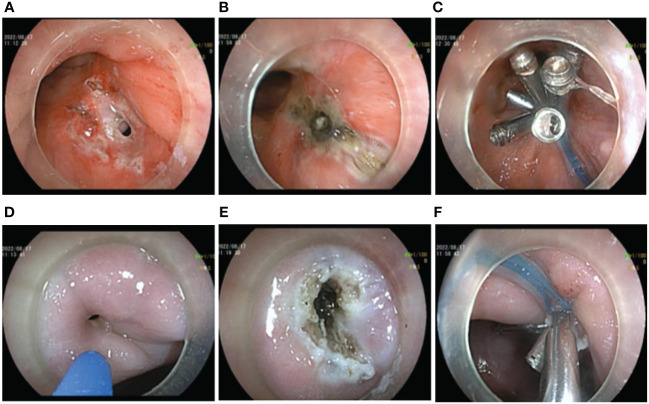
Operation procedure by transanal and transvaginal endoscopy. **(A–C)** Transanal endoscopic repair of rectal fistula. **(A)** Preoperative endoscopic creation of mucosal pinhole. **(B)** APC electrocauterization. **(C)** Rectal fistula clamped by a “purse string suture” clip with nylon ring-assisted TTSC. **(D–F)** Transvaginal endoscopic vaginal fistula repair. **(D)** Preoperative situation of vaginal fistula. **(E)** APC electrocauterization. **(F)** Vaginal fistula clamped by a “string suture” clip with nylon ring-assisted TTSC.

A transanal gastrointestinal endoscopic repair of the rectal fistula was performed in the left lateral decubitus position while awake. Firstly, the endoscope was inserted into the stoma of rectal fistula and a mucosal pinhole was created by incising the scarred mucosal layer with a hook knife (FUJIFILM BL-7000, FUJIFILM EG-580RD, Knife It, OLYMPUS KD-611L, DualKnife OLYMPUS KD-655L). This step minimized the negative effects of the pre-existing sclerotic tissue and fibrotic tissue on-clip anchoring in the fistula. Simultaneously, the fistula mucosal tissue and the fistula orifice were electrocauterized by APC (current flow: 1.2 L/min; 40 W; ERBE, VIO 200D, Germany) to promote tissue regeneration and facilitate mucosal healing and repair within the fistula cavity. The TTSC was inserted through the incision hole and the fistula was clamped along the contralateral direction, then the fistula was completely clamped with a nylon ring assisted by the TTSC “purse-string suture” (**Figures 2A–C**). In the latter stage of the procedure, the vaginal fistula was repaired by transvaginal gastrointestinal endoscopy of the patient while in the left lateral decubitus position. A sterilized gastrointestinal endoscope was inserted into the vaginal fistula. Similarly, the fistula scar mucosal tissue and the mucosa of the fistula margin were cauterized by APC, and the vaginal fistula was closed by a “purse-string suture” with a nylon loop-assisted TTSC (**Figures 2D–F**). A total of 24 endoscopic clips were used, and the total operation time was 125 min.

**Figure 3:**
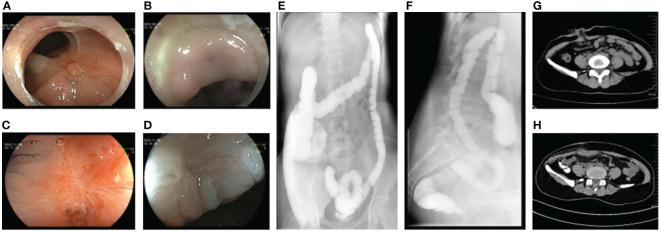
Postoperative auxiliary examination of the patient. **(A)** Endoscopic manifestations of rectal fistula 1 month after repairing operation. **(B)** Endoscopic manifestations of vaginal fistula 1 month after repairing operation. **(C)** Endoscopic manifestations of rectal fistula 3 months after repairing operation. **(D)** Endoscopic manifestations of vaginal fistula 3 months after repairing operation. **(E, F)** No leakage of barium enema contrast medium out of the rectal lumen. **(G)** Status of ileostomy. **(H)** Status post-ileostomy.

The patient was observed without postoperative complications, and no unexpected excretions were discharged from the vagina. The patient was discharged from the hospital on the seventh postoperative day. At the follow-up visit after 5 weeks, a reexamination by endoscopy was performed to observe the defective area. The fistula site was in a healing state with visible fresh healing tissues without air blowing into the vagina (**Figures 3A, B**). Another endoscopic examination 12 weeks later revealed white scar-like changes in the mucosa at both the rectal and vaginal fistula sites (**Figures 3C, D**), indicating a good prognosis. A barium enema was performed, revealing no contrast outside the rectal cavity (**Figures 3E, F**). Gynecologic examination showed only postoperative changes in the vaginal wall with no evidence of fistula. As the repair was satisfactory, the ileostomy was subsequently closed (**Figures 3G, H**) and the patient recovered without incident. During the 1-year follow-up, the patient had no symptoms of recurrence, had no discomfort, and had normal sexual function.

## 4 Discussion

Clinically, rectovaginal fistula is a rare complication after PPH combined with injection sclerotherapy for hemorrhoids, which is usually treated medically and surgically, with surgical approaches being more preferable. However, the success rate of fistulae closure is less than 50% due to the infection caused by the presence of sclerosis or fibrosis in the fistula and its surrounding intestinal walls (1). The surgical approach depends on the complexity of the fistula and the presence of associated sphincter damage. Options include transvaginal, transrectal, transperineal, or transabdominal (cesarean or laparoscopic) approaches, while simultaneous anal and vaginal repair of fistulae has rarely been reported. However, our case study presented a new endoscopic procedure combining a transanal and vaginal repair of the fistula. Notably, it was controversial whether a stoma should be made in the treatment of rectovaginal fistula. Given that the patient’s fistula was large and complex and the infection status of the stoma was serious, we chose to perform preventive ileostomy to create a cleaner operative area. Surgical repair was performed when the vagina no longer had fecal outflow. The stoma was closed as soon as the operative wound has healed (3).

In particular, owing to the injection of submucosal sclerosing agents and the formation of a scar in the rectal mucosa, the tissue around the fistula became stiffer and tighter, which caused difficulties in anchoring; in addition, the repaired and healed mucosa in the fistula cavity was not conducive to the closure of the fistula. Some case reports have described the preoperative endoscopic use of a needle knife to create a mucosal pinhole by electrocautery, followed by electrocauterization of the fistula tissue and insertion of a clip pin into the incision hole to achieve a tight closure of the fistula (4). It has also been reported that electrocauterization combined with clip closure via APC can facilitate the fusion of juxtaposed tissues and has been successfully applied to many types of gastrointestinal fistulae (4–6). Therefore, in our case, not only was the mucosal pinhole created endoscopically by a hook knife, but the healed and repaired mucosal tissue and the orifice tissue in the fistula were electrocauterized by APC as well. Traumatic inflammatory reaction and ulceration were formed in the fistula cavity, which promoted the regeneration of mucosal tissue, and facilitated the fusion of parallel tissues and sealing the fistula for better repair. In addition, the choice of endoscopic clamp was also very important. Currently, there are two types: through-the-scope clip (TTSC) and over-the-scope clip (OTSC). OTSC is considered to be effective in closing wounds and defects with a diameter of less than 20 mm, but it is expensive, while TTSC is often used for perforations with a diameter of less than 10 mm. If the perforation diameter is large, a variety of devices can be selected to complete the closure together. Thus, the combined application of nylon ring and metal clamp is an important and creative technology. Moreover, a large, multicenter, retrospective study on the use of TTSC for the treatment of gastrointestinal fistulae showed that the success rate of fistula repair was 42.9% (7), while studies on the short-term results of OTSC in the treatment of recurrent RVF have shown that the success rate and cure rate are 26% and 20%, respectively (2). In view of this, from the perspective of economy, fistula size, and fistula repair success rate, we chose nylon ring-assisted TTSC for fistula suture treatment. In this case, the fistula was completely clamped with a nylon loop-assisted TTSC followed by the anchoring of the marginal mucosa of the fistula, as well as the electrocauterization of the orifice and mucosa in the fistula. Moreover, vaginal endoscopy was performed and the same method was applied to repair the vaginal fistula. Our novel endoscopic approach achieved complete closure of the fistula in a single treatment, reducing the risk of recurrence. The procedure was very effective in safely and permanently closing the fistula without complications. The involved techniques such as the anchoring at the marginal mucosa of the fistula, the electrocauterization of the orifice and mucosa in the fistula, and the “purse-string suture” clamping by nylon loop-assisted TTSC altogether formulated a new approach for the treatment of rectovaginal fistulae with large orifices.

## 5 Conclusion

Our novel operative technique combined transanal endoscopy with vaginal endoscopy to cure this patient with rectovaginal fistula, involving the preoperative endoscopic creation of a mucosal pinhole, APC electrocauterization, and “purse-string” clamping with nylon loop-assisted TTSC. This technique achieved complete closure of the fistula in a one-off treatment, which consequently reduced the risk of recurrence without any complications. Hence, such a technique turned out to be a safe and minimally invasive surgical approach for the treatment of refractory RVF with large fistulae, which should be taken into consideration during clinical treatment.

## Data availability statement

The original contributions presented in the study are included in the article/supplementary material. Further inquiries can be directed to the corresponding authors.

## Ethics statement

The studies involving humans were approved by Ethics Committee of the First People’s Hospital of Yunnan Province (Approval No. KHLL2023-KY119). The studies were conducted in accordance with the local legislation and institutional requirements. The participants provided their written informed consent to participate in this study. Written informed consent was obtained from the individual(s) for the publication of any potentially identifiable images or data included in this article.

## Author contributions

TH: Data curation, Writing – original draft, Writing – review & editing. WZ: Data curation, Funding acquisition, Writing – original draft, Writing – review & editing. N-fM: Writing – review & editing. XB: Writing – original draft. LZ: Writing – review & editing. K-lY: Writing – original draft. G-qY: Writing – review & editing. C-mR: Ethics approval application, Writing – review & editing. JW: Data curation, Writing – review & editing. PW: Funding acquisition, Project administration, Writing – original draft, Writing – review & editing. QG: Project administration, Writing – original draft, Writing – review & editing. ZZ: Formal Analysis, Funding acquisition, Writing – original draft, Writing – review & editing.
